# Why patients fail to seek information on OTC product interactions with a direct-acting oral anticoagulant: perspectives on information-seeking

**DOI:** 10.1186/s12875-025-02740-1

**Published:** 2025-02-21

**Authors:** Marley P.D. Magee, Janice B. Schwartz, Amanda McArthur, Ruey-Ying Liu, Derjung M. Tarn

**Affiliations:** 1 Department of Family Medicine, David Geffen School of Medicine at UCLA, University of California, Los Angeles, 10880 Wilshire Blvd., Suite 1800, Los Angeles, CA 90024 USA; 2 Departments of Medicine, Bioengineering & Therapeutic Sciences, University of California, San Francisco, San Francisco, CA USA; 3 Johns Hopkins University School of Medicine, Baltimore, MD USA; 4 Department of Sociology, National Chengchi University, Taipei, Taiwan

**Keywords:** Over-the-counter drug, Information seeking behavior, Drug interactions, Dietary supplements, Apixaban

## Abstract

**Background:**

Many patients taking direct-acting oral anticoagulants (DOACs) also consume over-the-counter (OTC) products (dietary supplements and OTC medications), yet many lack knowledge of potential interactions that may increase or decrease DOAC efficacy and may not seek information about OTC products. The objective of this study was to describe patient attitudes and beliefs that inhibited information seeking about potential apixaban-OTC product interactions.

**Methods:**

Participants included English-, Spanish-, Mandarin-, and Cantonese-speaking adults from two large academic medical centers who reported taking apixaban (a frequently prescribed DOAC) in the past month. Thematic analysis was performed on semi-structured interviews.

**Results:**

Sixty patients aged 24–93 years (mean = 65.3; SD = 15.6) were interviewed; 55% were women. Participants took a total of 236 OTC products. Those with potential interactions with apixaban warranting consideration for therapy modification included: ibuprofen (*n* = 14; 5.9%), aspirin (*n* = 8; 3.4%), and naproxen (*n* = 3; 1.3%). Interviews revealed 5 major themes related to a lack of information-seeking about OTC products: (1) patients lack awareness of the potential for interactions; (2) patients believe that OTC products are safe and/or regulated (largely because they were familiar with the products, had previously taken them, or assumed that dietary supplements were regulated by the Food and Drug Administration); (3) patients believe that providers are responsible for alerting patients about potential interactions (as patients assumed that providers were aware of their OTC product use); (4) patients had prior knowledge of and/or used OTC products infrequently; and (5) obtaining information can be inconvenient. Inquiries regarding preferred information sources revealed 59 (98.3%) patients most frequently sought or would seek information from physicians and 34 (56.7%) from the internet.

**Conclusions:**

Patients taking apixaban raised reasons for not seeking information about potential OTC product interactions that included poor awareness, perceptions regarding the safety of OTC products, and beliefs in provider responsibility for informing them about interactions. Greater patient education is needed regarding the potential for OTC product-DOAC interactions and the regulation of OTC products, particularly dietary supplements.

**Supplementary Information:**

The online version contains supplementary material available at 10.1186/s12875-025-02740-1.

## Background

Direct-acting oral anticoagulants (DOACs) are currently recommended for reducing the risk of stroke and systemic embolization in patients with non-valvular atrial fibrillation [1–6]. Additionally, DOACs are recommended for the treatment of deep venous thrombosis and pulmonary embolism, and for prophylaxis of deep venous thrombosis, which may lead to pulmonary embolism, in patients who have undergone hip or knee replacement surgery [1–6]. DOACs have thus largely replaced traditionally-used vitamin K antagonists such as warfarin [7, 8]. DOACs have several advantages over warfarin, including simplified dosing regimens, fewer food or medication interactions, and generally no need for regular monitoring [9–12]. Though DOACs have fewer interactions than warfarin [13, 14], commonly used over-the-counter (OTC) antiplatelet agents, such as aspirin and nonsteroidal anti-inflammatory drugs (NSAIDs) [15], and dietary supplements with antiplatelet activity, such as ginkgo biloba, turmeric, and ginger [16–18], can increase the risk of bleeding when taken with a DOAC. In contrast, DOACs metabolized by the CYP3A4 enzymes may have concentrations and efficacy decreased by inducers of these enzymes such as phenytoin, carbamazepine, and St. John’s Wort (a dietary supplement) [15–18]. 

Our previous survey of 771 patients taking apixaban, the most frequently prescribed DOAC [19], showed that 98% regularly took OTC products (dietary supplements containing one or more vitamins, minerals, herbs or other botanicals, amino acids or other substances used to supplement the diet [20] and/or OTC medications) and that one-third of these products had potentially serious interactions with apixaban [21]. One-third of the patients reported not informing their providers about their OTC product use [21]. Whether or why patients did not seek information about potential interactions with apixaban was not ascertained. Overall, the literature on patient information seeking about potential medication-OTC product interactions is sparse, with studies in this area focusing largely on the need for patient education [22, 23]. Studies suggest that patients may read written medical information about their prescription medicines but are not likely to actively seek information [24]. Yet knowledge is lacking about barriers to patient information-seeking about potential interactions. The objective of this study was to describe patient attitudes and beliefs that inhibit active information seeking about potential apixaban-OTC product interactions.

## Methods

### Study design, setting, participants

We conducted one-time semi-structured interviews with participants recruited from University of California, Los Angeles (UCLA) and University of California, San Francisco (UCSF) Health systems in the United States. Participants were purposively sampled to achieve racial/ethnic diversity and to ensure equal representation of those who had previously taken and not taken warfarin.

Potential participants were identified through large-scale electronic health record (EHR) data extractions at UCLA and UCSF. These data extractions yielded lists of patients aged 18 and older who were prescribed apixaban from July 2016–February 2017. Patients were emailed or sent postal mail invitations to participate in the study and were given an opportunity to opt-out. They were informed that the study focused on how often and why patients take over-the-counter drugs and dietary supplements with apixaban. Non-respondents received up to 2 follow-up telephone calls. A research associate screened those expressing interest for eligibility. Eligible patients were required to be 18 years of age or older, taking apixaban, and proficient in English, Spanish, Mandarin or Cantonese. To ensure diversity in our sample population we sought to recruit approximately 10–15 participants in each of 4 different racial/ethnic groups (Asian, Black, Hispanic, and White). Of 60 total participants, 55 (92%) used OTC products.

### Data collection

English-language interviews were conducted via telephone by a medical sociologist (AM) in 2017. Two undergraduate students, who were trained and closely supervised by investigators AM and DMT (a physician-investigator with qualitative research expertise), conducted interviews in Spanish, Mandarin, and Cantonese. Interview guide development was based on the investigators’ clinical expertise and on conceptual models of information-seeking [25, 26]. Interview questions asked about participant perceptions regarding taking OTC products in conjunction with apixaban, information-seeking about potential OTC product-apixaban interactions, discussions with their providers about OTC product use, and their preferences for sources of information about interactions **(**see **Appendix** for interview guide**)**. Participants also provided information on their demographics and OTC product use. Interactions were categorized based on Lexicomp and Natural Medicine database recommendations for OTC product use with apixaban. Interviews lasted about 30–45 min and were audio recorded. Participants provided verbal informed consent and received a $35 gift card for participation.

### Qualitative analyses

Interviews were transcribed verbatim and translated by a professional transcription company using standard procedures. Spanish, Mandarin, and Cantonese interview translations were verified for accuracy by the interviewers. Two coders, AM and DMT, each inductively analyzed interviews to develop codes related to patient information-seeking. DMT and MM (an undergraduate biology student) subsequently employed thematic analysis to develop themes describing patient attitudes and beliefs regarding information-seeking about OTC products. This process involved grouping related codes to create preliminary themes [27–29]. MM and DMT discussed preliminary themes and came to consensus; they reviewed these themes with the other investigators for external validity [27–29]. Based on consensus, the investigators determined that theoretical saturation (no emergence of new codes or themes from the data) was reached [20, 22]. ATLAS.ti 23 (Scientific Software Development, Berlin, Germany) was used for all analyses.

## Results

Sixty patients participated in the study. Their mean age was 65.3 years (SD = 15.6; range 24–93), with 33 (55%) being female, and 18 (30%) each identifying as Asian or Hispanic **(**Table 1**)**.

**Table 1:** Patient characteristics; *n* = 60

Characteristics	*n* (%) or mean (SD)
Age, y; mean (SD); range	65.3 (15.6); 24–93
Female; n (%)	33 (55)
Race/Ethnicity; n (%)	
Asian	18 (30)
Black	12 (20)
Hispanic	18 (30)
White	9 (15)
Other	3 (5)

Participants reported taking a total of 236 OTC products; 55 of 60 (91.7%) participants took at least one OTC product. The most commonly reported was vitamin D/calcium (*n* = 35; 14.8%) **(**Table 2**)**. Of all reported OTC products, those warranting consideration for therapy modification when taken with apixaban included ibuprofen (*n* = 14; 5.9%), aspirin (*n* = 8; 3.4%), and naproxen (*n* = 3; 1.3%), while an additional 23 warranted monitoring of the therapy (fish oil, ginkgo biloba, bismuth subsalicylate).

**Table 2:** OTC products taken; *n* = 236

OTC Product	*n* (%)
**Lexicomp: Consider therapy modification** [30]	
Ibuprofen	14 (5.9)
Aspirin	8 (3.4)
Naproxen	3 (1.3)
**Lexicomp: Monitor therapy** [30]	
Fish Oil*	18 (7.6)
Ginkgo Biloba^†^	3 (1.3)
Bismuth subsalicylate	2 (0.85)
**Lexicomp: No known interactions** [30]; **Natural Medicines: Be cautious with this combination (moderate interaction)** [31]	
Garcinia Cambogia	3 (1.3)
Melatonin	3 (1.3)
Turmeric	3 (1.3)
Niacin	1 (0.42)
**Lexicomp: No known interactions** [30]; **Natural Medicines: Be watchful with this combination (minor interaction)** [31]	
Vitamin D/Calcium	35 (14.8)
Magnesium	5 (2.1)
**Lexicomp: Data unavailable** [30]; **Natural Medicines: Be cautious with this combination (moderate interaction)** [31]	
Astaxanthin	2 (0.85)
Vitamin E	2 (0.85)
Aloe vera	1 (0.42)
Cranberry pill	1 (0.42)
DHEA	1 (0.42)
Digest Assure	1 (0.42)
Echinacea	1 (0.42)
**Variable depending on specific ingredients** [31]	
Multivitamin	24 (10.2)
**No known interactions (Lexicomp and/or Natural Medicines)** [30, 31]	
Other vitamins/minerals^‡^	29 (12.3)
Acetaminophen	28 (11.9)
Other non-vitamin non-mineral supplements^§^	21 (8.9)
Antihistamines/decongestants	9 (3.8)
Cough and cold medications	6 (2.5)
Antiulcer agents	10 (4.2)
Laxatives (MiraLAX, Ex-lax)	2 (0.85)

*NM: be watchful with this combination

^†^NM: be cautious with this combination

^‡^Vitamin C (*n* = 12), vitamin B (*n* = 8), biotin (*n* = 4), zinc (*n* = 2), iodine (*n* = 1), vitamin A (*n* = 1), vitamin K (n=1)

^§^glucosamine (*n* = 4), herbal tea (n=3), herbs (unspecified) (*n* = 3), lysine (*n* = 3), probiotic (n=2), chondroitin (*n* = 1), cinnamon (*n* = 1), Co-Q10 (*n* = 1), lactase (*n* = 1), lutein (*n* = 1), red yeast rice (*n* = 1)

Semi-structured interviews revealed 5 themes representing attitudes and beliefs related to a lack of active information-seeking regarding OTC products. Themes are described in greater detail below and additional examples are provided in Table 3.

**Table 3:** Themes and illustrative quotes related to information seeking about OTC product-apixaban interactions

	Themes Related to Lack of Information Seeking
**Lack of Awareness of the Potential for Interactions**	(On how likely they are to wonder about potential interaction) “Not at all. Not until now. You guys have got me thinking about it…I just didn’t realize there were, I didn’t realize there were contraindications…I think normally I don’t think of [OTC products] as interfering.” [P31]
**Belief that OTC Products are Safe and/or Regulated**	**Belief that OTC products are safe** “Over-the-counter products typically have fewer side effects. Fatal problems, deathly problems, or big problems are very, very rare. So I don’t evaluate it. If it’s like fish oil pills or multivitamins, we take them once in a while but don’t evaluate them.” [P46] “I think [dietary supplements are] natural, and I don’t see why they would harm me in any way.” [P26] **Belief that OTC products are regulated** “I would assume it’s safe to take because America has a good food and drug program. It wouldn’t be that severe if it had not passed the drug regulations.” [P05]
**Belief that Providers are Responsible for Alerting Patients about Interactions**	If it’s very serious, then [my doctor] will say something. He will tell me that this drug will interact with that drug, will cause it to not have its function. He will say it. If he doesn’t say anything, it’s most likely that there wouldn’t be serious side effects.” [P48] “The greatest thing that kept me from trying to get information is just kind of ignorance, you know, of not realizing that it’s incumbent on me to get information about a potential conflict. So, I would say ignorance of the need to do that, and then, the second thing would be just trust and confidence in the doctors that, if there was something there, they would tell me about it. Probably a combination of both.” [P59]
**Prior Experience and Infrequent Use Preclude Information-Seeking**	**Prior experience with OTC products** “I’ve never had any adverse reaction to taking over-the-counter medication, so I generally think that it is safe to do while following the specific directions of the medication.” [P14] “I’ve taken things like Advil before, and I’m familiar with them, and I wouldn’t feel the need [to seek information about them].” [P08] **Infrequent OTC product usage** (On what kept them from talking to their doctor about an OTC product) “I don’t take [Aleve] that often, so I don’t feel like it’s necessary.” [P06] **Experience taking OTC product w/ another anticoagulant makes them sure of effects** “I talked to my primary doctor before I started - or after I took the Warfarin. And since then, I can see the Tylenol, that’s a safe medication for me if I want to take over-the-counter combined with my blood thinner.” [P37]
**Inconvenience of Obtaining Information**	(On what might keep them from talking to a healthcare provider before taking an OTC product) “Just east of access…it takes me like three months to get an appointment with [my doctor].” [P07]

## Theme 1: patients lack awareness of the potential for interactions

A common theme among participants centered on a lack of awareness regarding potential OTC product-apixaban interactions. For example, when asked to reflect about the possibility of interactions, one participant noted: “It just isn’t in my mind. I never thought about conflict between the two.” [P36] Unsurprisingly, these participants noted that they did not actively seek information about potential interactions.

Conversely, a handful of participants who were aware of the potential for interactions felt obliged to do further research. One explained that they wondered about interactions “[every] single time” they took an OTC product and claimed, “I’m pretty stressed out about whether or not something will happen to be detrimental to my health…based on an interaction or side effect.” [P14] This awareness of interactions led the participant to search the internet for information prior to taking nearly all their OTC products.

## Theme 2: patients believe that OTC products are safe and/or regulated

A major barrier to information-seeking was the belief that all OTC products are “safe,” with some participants bolstering their views of safety by stating that these products are regulated by the Food and Drug Administration (FDA). Participant comments about safety and FDA regulation focused mostly on OTC medications, but many believed that dietary supplements were safer than OTC medications because they are natural: “I generally think of…vitamins as being more benign, so generally less potential for risk, whether that’s from drug interaction or anything.” [P28].

On the other hand, participants who were aware that OTC products are not regulated in the same way as prescription medications were more likely to actively seek information about interactions. For example, one participant stated they would seek information because: “I know [OTC products] are not necessarily regulated and they could interfere with my prescription medication.” [P02] Several participants who understood that apixaban was a “new” medication were more cautious and actively sought information about potential interactions.

## Theme 3: patients believe that providers are responsible for alerting patients about interactions

Many participants assumed that their providers would inform them of any potential OTC product-apixaban interactions, thus reducing the need for them to further inquire about interactions. One participant said: “[M]y doctors have [not] indicated that any of these other things are a problem…I rely on my doctors to tell me if it’s advisable or not.” [P35] Occasionally these participants added that their providers already knew what OTC products they were taking. In one participant’s words, “[My doctors] know what I’m taking. So therefore, they would tell me, the doctors I have. I have that much confidence in them.” [P06].

Conversely, participants who felt responsible for their medications were more motivated to seek information. One participant explained, “It’s better to be an educated consumer than an unknowing consumer.” [P02].

## Theme 4: patients had prior knowledge and/or used infrequently

Regardless of whether participants previously took an OTC product concomitantly with apixaban, they often cited their prior experiences and familiarity with OTC products as a reason for their lack of information-seeking. One participant said, “I took Tylenol or ibuprofen for years, and other than knowing that I had to take it with food, I didn’t even think about it, so probably [my prior usage] would affect [my information-seeking] a lot.” [P31] In contrast, participants who felt that they lacked knowledge about an OTC product were motivated to seek information about it. As one participant reflected, they more were likely to wonder about potential interactions if “it was something I never had before, like an [OTC product] that I’d never used before.” [P17].

The absence of prior adverse reactions was often viewed as justification for the use of OTC products. For example, one participant said there was no cause for alarm because “everything over-the-counter, if you don’t have a problem…that means you will be okay.” [P37] However, those who had previously experienced OTC product-apixaban interactions were more wary of interactions with other OTC products and noted that they were more likely to seek information about interactions.

A few participants who had previously taken an OTC product with a different anticoagulant did not feel the need to seek information because they believed that they already knew about potential interactions. On the other hand, a small number of participants who previously took warfarin were hesitant to take OTC products without seeking information, even if they had not previously experienced interactions.

Some participants believed that infrequent OTC product use precluded interactions with apixaban. As one participant noted, they did not disclose their acetaminophen use to their provider “because I’m not a person to regularly take Tylenol.” [P13] Conversely, regular OTC product use was more likely to promote information-seeking. One participant reflected that they would seek information if “I had to take something for longer than one day, so if there was any length of time involved.” [P17]Similarly, another indicated that they would ask their provider about acetaminophen but not ibuprofen because “I might take ibuprofen…every 2 months, whereas I might take Tylenol 2–3 times a week.” [P10]. 

## Theme 5: obtaining information can be inconvenient

For many, poor information access was an issue. For example, participants were less motivated to seek information about potential interactions if they needed to schedule an appointment with their provider. As one participant said about not talking to their provider about their OTC product use: “it was just inertia.” [P23] On the flipside, increased accessibility of information promoted information seeking. Several participants stated that it was easy to find information on the internet: “I look on Google. And I would look up the [OTC] product and take what different pharmaceutical companies have to say about it.” [P05].

Many participants preferred to receive information passively rather than actively seeking information. One participant noted: “I would have to hear some news about [the OTC product] or read it. And not go looking for the news. The news drops on me.” [P16] Receipt of passive information about potential interactions could promote active information-seeking. For example, one participant said they would be “[extremely] likely” to seek information because the “warning on the Eliquis bottle” led them to believe that apixaban is “sensitive to other over-the-counter supplements.” [P17] However, a minority noted that passive information regarding their apixaban prescription was not always helpful because they felt overloaded with information. One participant expressed difficulty reading the information packet that came with their prescription: “Yeah, the big sheet, and it has a bunch of pages on it…I mean, just simplify it so we don’t have to go through all that.” [P27].

### Participant feedback on information sources

**Fig. 1:**
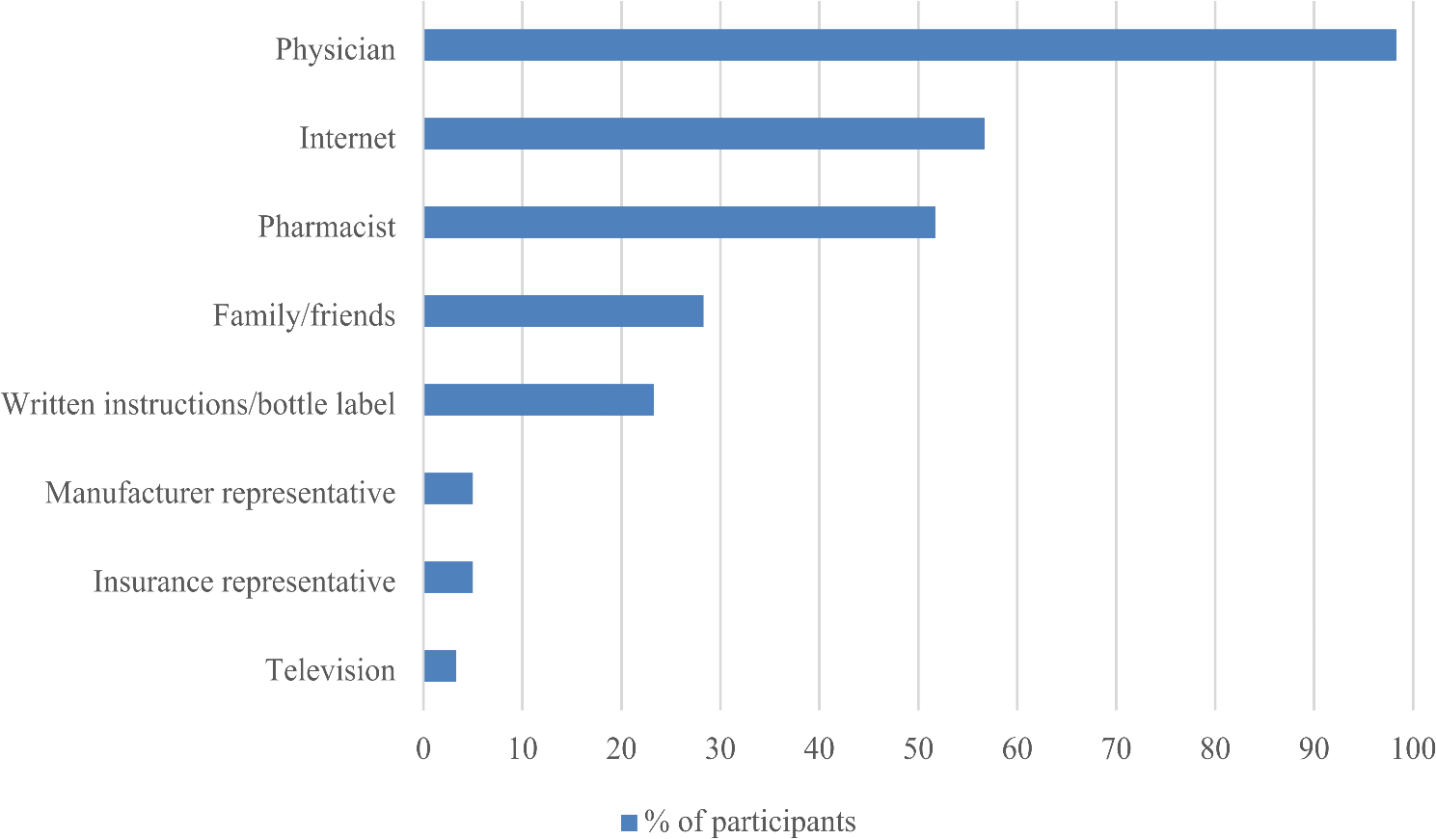
Preferred Information Sources; *n* = 60

In addition to the themes identified above, participants were asked to discuss the information sources they had previously utilized or would consider using if they decided to seek information. These sources ranged from conversations with others (including family, friends, physicians, pharmacists, insurance representatives, and drug manufacturer representatives) to seeking information through alternative channels. Thirty-eight participants (63.3%) used a combination of interpersonal interaction and other sources, twenty-two (36.7%) preferred interpersonal interaction exclusively, while none solely preferred sources other than speaking with a person **(**Fig. 1**)**. The healthcare providers most commonly preferred for seeking information included primary care clinicians (56.7% of participants), pharmacists (51.7%), and cardiologists (41.7%).

Other preferred sources of information included the internet (56.7%), written information given with a prescription or on prescription bottles (23.3%), and public information (e.g., television and online advertisements) (3.3%). Internet sources cited included the Mayo Clinic, Centers for Disease Control, National Institute of Health, National Institute of Mental Health, WebMD, Wikipedia, UpToDate, university websites, drug manufacturers, and unspecified scientific articles.

## Discussion

Semi-structured interviews with patients taking apixaban, a commonly prescribed DOAC, revealed that information-seeking about OTC product-apixaban interactions was inhibited by several key perceptions about OTC products: (1) a lack of awareness of apixaban-OTC product interactions, (2) the perception that OTC products were safe and/or regulated, (3) the belief that providers bore the responsibility for alerting patients about OTC product-apixaban interactions, and (4) previous experiences of non-problematic or infrequent OTC product use. The findings are consistent with our prior study, which demonstrated that about two-thirds of patients taking apixaban lacked knowledge about the risk for potential increased bleeding when combining apixaban with an NSAID [21]. For many patients in this study, the potential for interactions never crossed their mind, suggesting that better patient education is needed. Indeed, some participants noted that knowledge about potential interactions spurred action towards accessing information to avoid potential interactions.

A common sentiment among study participants was that potential interactions with apixaban were of no concern if they had previously used an OTC product without any problems (even if it was not taken concomitantly with apixaban). Similarly, if they were familiar with an OTC product or if they used it infrequently, there was no need to worry about interactions. As commonly used OTC products such as NSAIDs can increase the risk of bleeding when taken with apixaban [32], increased awareness about these risks is clearly needed. Consultations about potential OTC product-apixaban interactions could be provided at the point of prescribing, at the pharmacy when apixaban is dispensed or when patients approach pharmacists for advice about OTC product use, or during follow-up office visits.

Contrary to the misconception expressed by many participants that OTC products are FDA-regulated, the FDA does not mandate safety or efficacy testing for dietary supplements [33], and there have been instances in which dietary supplements were found to contain contaminated ingredients, including active pharmaceutical ingredients [30]. Our study reinforced findings in the literature showing that patients have misperceptions about the safety of dietary supplements [31, 34]. These data highlight the need for greater patient education about the FDA’s oversight of the safety and efficacy of dietary supplements. In addition, participants often equated safety with the absence of interactions, suggesting that providers may need to pay greater attention to educating patients about the possibility of interactions, even with OTC medications perceived as ‘safe’.

Some study participants were aware of potential interactions with apixaban but did not actively seek information about them because they believed it was their provider’s responsibility to alert them about any potential interactions. Disclosure of OTC medication use to providers has not been well-documented, but studies have shown that patients often neglect to inform their providers about their dietary supplement use [35], communication about dietary supplements is often sparse [36], and documentation is often lacking [37]. Efforts to ask patients specifically about both their OTC medication and dietary supplement use would likely increase patient disclosure of their use of these products [35] and documentation of their use in EHRs could leverage the potential for EHR systems to identify possible interactions.

The current study has some limitations. All participants were recruited from large academic health systems, and while our participants were a racially/ethnically diverse sample, this was a qualitative study with small numbers of patients in each group, thus precluding comparisons between the groups. Additional work is needed to investigate whether racial/ethnic disparities in information-seeking behaviors exist. This study did not collect participant educational level or health literacy, which may have affected participants’ information-seeking behaviors. It is possible that awareness of potential apixaban-OTC product interactions has increased since these data were collected in 2017; nonetheless, existing literature on patient information-seeking concerning drug interactions is still limited.

## Conclusions

In conclusion, there is a pressing need for improving patient awareness of potential interactions between prescription medications and OTC products, particularly with anticoagulants such as DOACs, given the increased risk of bleeding or decreased medication efficacy [15, 18]. Key barriers to patient information-seeking include poor patient awareness of the possibility for interactions, beliefs about the safety of OTC products (particularly dietary supplements), and assumptions that providers are aware of their OTC product use and would alert them to potential interactions. Both providers and pharmacists could take greater responsibility for patient education. However, providing only written material without discussion may not be adequate. While written materials from providers and pharmacists could supplement counseling, passive distribution of information may overwhelm or confuse patients and lead to limited retention. Further, patients obtaining written information online, out of the context of a pharmacist encounter or provider visit, may require guidance to evaluate the reliability of this information. Gaps in patient knowledge regarding potential interactions may not be limited to apixaban; further research is warranted to explore patient awareness of potential interactions with other anticoagulants and medications, specifically those that are similarly metabolized by CYP3A4 enzyme.

## Electronic supplementary material

Below is the link to the electronic supplementary material.

Supplementary Material 1

## Data Availability

No datasets were generated or analyzed for this study. This study’s data consist of transcripts of audio recorded interviews. These data are available from the last author upon reasonable request and with IRB approval.

## Abbreviations

DOAC: direct-acting oral anticoagulant
OTC: over-the-counter
UCLA: University of California, Los Angeles
UCSF: University of California, San Francisco
EHR: electronic health record
IRB: institutional review board
FDA: Food and Drug Administration
NSAID: nonsteroidal anti-inflammatory drug
