# Supplementary material for: Why patients fail to seek information on OTC product interactions with a direct-acting oral anticoagulant: perspectives on information-seeking
Source: BMC Prim Care. 2025 Feb 21;26:47. doi: 10.1186/s12875-025-02740-1 (PMC11846430; doi:10.1186/s12875-025-02740-1)
Supplement: Supplementary file 1 — Supplementary Material 1 [file 12875_2025_2740_MOESM1_ESM.docx]

**Appendix**

**Major Semi-Structured Interview Questions**

| Introductory question | Approximately how long have you been taking Eliquis?  Since you started taking Eliquis, what over-the-counter medications or dietary supplements have you taken? |
| --- | --- |
| Perceptions about safety/interactions with apixaban | When you think about taking an over-the-counter medication or dietary supplement, how likely are you to wonder about whether it might cause problems with your Eliquis?  Imagine that you’re combining Eliquis with an over-the-counter medication such as advil, motrin, or aspirin. In general, what kinds of problems (if any) do you believe you might have?  I’d like you to imagine that you’re combining Eliquis with a dietary supplement, such as vitamins, fish oil, and herbal supplements. In general, what kinds of problems do you believe you might have? |
| Information-seeking about interactions before taking OTC product | Given that you’re taking Eliquis, how likely are you to try to get information about whether an over-the-counter product is safe to take?  [If you wanted to find information], where are you most likely to go for information about potential problems with combining Eliquis with an over-the-counter product? (e.g., talk to somebody, internet, ads)  While you’re taking Eliquis, how likely are you to talk to a healthcare provider like a doctor, nurse, or pharmacist before taking an over-the-counter product?  Imagine that you’re trying to decide about whether to take an over-the counter product. What types of information would best help you decide whether to take it product while you’re on Eliquis? |
| Provider discussion/disclosure before and after taking OTC product | (If OTC product started before taking Eliquis) How much did you talk to any healthcare provider about [OTC product name] after you started taking Eliquis/ [OTC product name]?  (If OTC product started after taking Eliquis) How much did you talk to a healthcare provider when you were thinking about taking [OTC product name] (before you actually took it)? |
